# Substrate stiffness can affect the crosstalk between adipose derived mesenchymal stem cells and macrophages in bone tissue engineering

**DOI:** 10.3389/fbioe.2023.1133547

**Published:** 2023-07-27

**Authors:** Zeyang Liu, Jin Liu, Jipeng Li, Yinwei Li, Jing Sun, Yuan Deng, Huifang Zhou

**Affiliations:** ^1^ Department of Ophthalmology, Shanghai Ninth People’s Hospital, School of Medicine, Shanghai Jiao Tong University, Shanghai, China; ^2^ Shanghai Key Laboratory of Orbital Diseases and Ocular Oncology, Shanghai Ninth People’s Hospital, School of Medicine, Shanghai Jiao Tong University, Shanghai, China

**Keywords:** substrate stiffness, ADSCs, macrophages, polarization, bone repair

## Abstract

**Purpose:** This study aimed to explore the effect of biomaterials with different stiffness on Adipose Derived Mesenchymal Stem Cells (ADSC)–macrophage crosstalk in bone tissue engineering and its role in bone repair.

**Methods:** Biomaterials with Young’s modulus of 64 and 0.2 kPa were selected, and the crosstalk between ADSCs and macrophages was investigated by means of conditioned medium treatment and cell co-culture, respectively. Polymerase chain reaction (PCR) and flow cytometry were used to evaluate the polarization of macrophages. Alkaline phosphatase (ALP) and alizarin red staining (ARS) solutions were used to evaluate the osteogenic differentiation of ADSCs. Transwell assay was used to evaluate the chemotaxis of ADSCs and macrophages. Moreover, mass spectrometry proteomics was used to analyze the secreted protein profile of ADSCs of different substrates and macrophages in different polarization states.

**Results:** On exploring the influence of biomaterials on macrophages from ADSCs on different substrates, we found that CD163 and CD206 expression levels in macrophages were significantly higher in the 64-kPa group than in the 0.2-kPa group in conditioned medium treatment and cell co-culture. Flow cytometry showed that more cells became CD163^+^ or CD206^+^ cells in the 64-kPa group under conditioned medium treatment or cell co-culture. The Transwell assay showed that more macrophages migrated to the lower chamber in the 64-kPa group. The proteomic analysis found that ADSCs in the 64-kPa group secreted more immunomodulatory proteins, such as LBP and RBP4, to improve the repair microenvironment. On exploring the influence of biomaterials on ADSCs from macrophages in different polarization states, we found that ALP and ARS levels in ADSCs were significantly higher in the M2 group than in the other three groups (NC, M0, and M1 groups) in both conditioned medium treatment and cell co-culture. The Transwell assay showed that more ADSCs migrated to the lower chamber in the M2 group. The proteomic analysis found that M2 macrophages secreted more extracellular remodeling proteins, such as LRP1, to promote bone repair.

**Conclusion:** In bone tissue engineering, the stiffness of repair biomaterials can affect the crosstalk between ADSCs and macrophages, thereby regulating local repair immunity and affecting bone repair.

## 1 Introduction

The stiffness and surface morphology of biomaterials are their inherent properties (Erica et al., 2020). They can affect the biological behavior of the cells attached to the surface and then influence the biological activity of biomaterials (Kyle and David, 2017), thereby endowing biomaterials with some unique functions. Studies showed that the stiffness of the biomaterials affected the normal physiological state of the cells by controlling the water and ion channels (Ming et al., 2017). Meanwhile, some other studies showed that substrate stiffness affected the direction of stem cell differentiation by regulating mTOR pathway activity (Jessica et al., 2018). These findings provided solid evidence that the inherent properties of biomaterials influenced the biological functions of biomaterials. Recently, a large number of researchers have been focusing on the point that the inherent properties of biomaterials also have an essential impact on the immune characteristics of biomaterials (Rukmani et al., 2019; Jefferson et al., 2021; Mainak et al., 2021; Zhuqing and Kaitlin, 2021). Current studies mainly investigated the influence of the surface topography of biomaterials on their immunological properties and biological functions (Joshua et al., 2021). However, the effect of stiffness on biomaterial immunological properties remains to be explored more deeply.

The immunological properties of the biomaterials depend on surface-attached cells to function, especially stem cells in tissue engineering (Francois et al., 2011; Ashkan and Anthony, 2017). ADSCs were undoubtedly the better choice for bone tissue engineering compared with mesenchymal stem cells, such as BMSCs and PSCs, which were crucial in the self-repair of bone defects (Baker et al., 2015; Naderi et al., 2017; Debnath et al., 2018). With the same capacity of osteogenic, adipogenic, and chondrogenic differentiation (Meliga et al., 2007), ADSCs have unique advantages, including wider source, larger quantity, and easy extraction (Won et al., 2017; Li et al., 2020; Shukla et al., 2020; Li et al., 2022), which make them the most widely used adult stem cells in research and clinical practice (Zavan et al., 2010; Przybyt and Harmsen, 2013; Joo et al., 2017; Lee et al., 2021; Nolan et al., 2021). ADSCs are also pioneers in the clinical translation of stem cells in bone tissue engineering and other immunomodulatory applications (Sayegh et al., 2019; Mazini et al., 2021; Li et al., 2022), so we chose ADSCs as the research object. At present, the research on ADSCs mainly focuses on the stemness maintenance and differentiation pathways of stem cells (Yuqin et al., 2014; Wei et al., 2015; Dandan et al., 2021), as well as the effects of biomaterials directly on the proliferation and differentiation of ADSCs (Chen et al., 2016; Lei et al., 2020; Xiao et al., 2021). However, the interaction between ADSCs and the immune microenvironment in bone tissue engineering remains to be investigated.

Immune cells, such as macrophages, neutrophils, and T Cells, are effector cells for biomaterials to exert immunological properties. Macrophages are the most important components of the bone repair immune microenvironment (Wynn et al., 2013). Macrophages act not only as *in situ* osteoclasts to remodel new bone in the bone defect site (Udagawa et al., 2021) but also as circulating macrophages migrating to the injury site and regulating the local immune activation state (Mantovani et al., 2013). The most important biological behavior in macrophage immune regulation is macrophage polarization (Locati et al., 2013). Macrophage polarization is mainly divided into two categories: M1 and M2, representing proinflammatory (Murray, 2017; Atri et al., 2018) and anti-inflammatory (Shapouri-Moghaddam et al., 2018; Yunna et al., 2020) properties, respectively. M1–M2 sequential polarization is essential in bone defect repair (Loi et al., 2016). Local macrophages mainly undergo M1 polarization in the early stage of the injury, mediating acute inflammatory responses to control infection and recruit many immune cells (Murray and Wynn, 2011; Mantovani et al., 2013; Wynn and Vannella, 2016). After the inflammation calms down, the macrophages in the injured area mainly turn to M2 polarization, secreting many factors that promote bone repair (Pajarinen et al., 2019), making them the key cells in bone regeneration (Gentek et al., 2014). Some recent studies investigated the polarization potential of macrophages adherent to biomaterials in bone tissue engineering (Yizhou et al., 2021). However, most macrophages were nonadherent and distributed in the injury microenvironment. In this case, the cohesion of adherent stem cells on macrophages is vital. Thus, we wondered whether the stiffness of biomaterials could interfere with the crosstalk between ADSCs and macrophages through the ADSCs adherent to the surface of biomaterials, thereby affecting the osteogenic differentiation of ADSCs and the polarization of macrophages, and ultimately affecting the result of bone repair.

This study aimed to explore the effect of biomaterials with different stiffness on the ADSC–macrophage crosstalk and its role in bone repair. We evaluated the polarization states of macrophages, the differentiation capacity of ADSCs, and their mutual influence on migration potential. Besides, we compared the secreted proteins of macrophages in different polarization states and ADSCs on different substrates, which provided a preliminary explanation of the mechanism of ADSC–macrophage crosstalk. We hope that the findings of this study will bring new insights into the effect of biomaterial stiffness on the immunological properties of biomaterials and their potential application in bone tissue engineering.

## 2 Materials and methods

### 2.1 Cell isolation, cell culture, and conditioned medium collection of ADSCs

Human primary ADSCs were obtained from the eyelid adipose tissue of patients. The study was approved by the independent ethics committee of the Shanghai Ninth People’s Hospital and the informed consent form has been signed. Briefly, the adipose tissue was cut into small pieces and digested with collagenase A for 8 h. The digested tissue was centrifuged for 15 min (1,200 rpm, 37°C). Then, the precipitate was resuspended in a complete culture medium (DMEM with 10% FBS and 1% penicillin/streptomycin, Gibco) and seeded into a 10-cm culture dish. The culture medium was changed every 3 days. The culture plates with different Young’s modulus of 64 kPa and 0.2 kPa (Advanced Biomatrix Co. Ltd., USA) were covered with collagen I (0.1%, Roche) for 30 min to collect the conditioned medium of ADSCs on different substrates. Then, ADSCs were seeded into plates at a density of 1.67 × 10^4^/cm^2^ (8 × 10^4^/mL). The medium was changed into DMEM without FBS after cell adhesion. The conditioned medium was collected 24 h later, filtered with a 0.22-μm filter, and stored at −80°C.

### 2.2 Cell culture, differentiation, polarization, and conditioned medium collection of macrophages

Human THP-1 cells were obtained from ATCC. The cells were cultured with a complete culture medium (RPMI-1640 with 10% FBS and 1% penicillin/streptomycin, Gibco), and the medium was changed daily. For macrophage differentiation, the THP-1 cells were suspended in a complete culture medium with PMA (100 ng/mL, Sigma) and seeded into a 10-cm culture dish at a density of 6.37 × 10^4^/cm^2^ (5 × 10^5^/mL). The THP-1 cells were stimulated overnight and then washed with phosphate-buffered saline (PBS) three times. The adherent cells were M0 macrophages. Human primary monocyte derived macrophages (MDMs) were obtained from patients underwent eye plastic surgery, which was approved by the independent ethics committee of Shanghai Ninth People’s Hospital and the informed consent form has been signed. Briefly, human peripheral blood was obtained and peripheral blood mononuclear cells (PBMCs) were isolated by density gradient centrifugation. Then CD14^+^ monocytes were isolated by magnet-activated cell sorting. Monocytes were suspended with complete inducing medium (IMDM with 10% FBS and 1% penicillin/streptomycin, Gibco; Human M-CSF, 50 ng/mL, Peprotech) and seeded into culture dishes at a density of 6.37 × 10^4^/cm^2^ (5 × 10^5^/mL). MDMs were obtained after 7 days of induction. For M1 polarization, the macrophages were cultured with LPS (1 mg/mL, Sigma) and IFN-γ (20 ng/mL, Peprotech) for 24 h. For M2 polarization, the macrophages were cultured with IL-4 (20 ng/mL, Peprotech) and IL-13 (20 ng/mL, Peprotech) for 24 h. The conditioned medium was collected after culturing the M0, M1, and M2 macrophages with RPMI-1640 without FBS for 24 h. Then, the supernatant was filtered and stored at −80°C.

### 2.3 Effect of ADSCs on the polarization of macrophages

#### 2.3.1 Conditioned medium treatment

The THP-1 cells were seeded into six-well plates and differentiated into M0 macrophages using the differentiation medium. ADSCs were seeded on different substrates including 64 kPa, 0.2 kPa and standard culture plates to collect conditioned medium. Standard culture plates inoculated with ADSCs were used as control. Then, the conditioned medium of ADSCs was prepared on 64-kPa and 0.2-kPa substrates and configured into a complete conditioned medium (conditioned medium +10% FBS, Gibco). It was added to M0 macrophages in different wells and incubated in a constant-temperature incubator for 48 h. Meanwhile, we prepared wells containing normal complete culture medium (normal culture medium +10% FBS, Gibco) as negative references, and wells treated with M1 and M2 polarizing solutions as positive references. All wells were incubated in a constant-temperature incubator for 48 h.

#### 2.3.2 Cell co-culture

The THP-1 cells were mixed with the differentiation medium and seeded into a Transwell chamber with a pore size of 0.3 μm; therefore, they could not enter the lower layer through the pores of the chamber. The chambers were then placed in a common 24-well plate containing the differentiation medium, and the cells were differentiated in a constant-temperature incubator for 24 h. The 64-kPa and 0.2-kPa culture plates were taken and inoculated with ADSCs after coating to make them adhere to the wall and reach 60% confluence. Standard culture plates inoculated with ADSCs were used as control. The Transwell chambers were briefly washed with a PBS solution and then placed in culture plates with varying stiffness for culturing ADSCs. Subsequently, the chambers were co-cultured in an incubator for 48 h. At the same time, a negative reference (well with normal culture medium) and a positive reference (two different polarizing solutions, M1 and M2) were prepared. MDMs underwent the same treatment with THP-1-derived M0 macrophages.

#### 2.3.3 RNA extraction and polymerase chain reaction

The macrophages and ADSCs were treated with a conditioned medium or cell co-culture. Macrophage samples were collected after polarization while ADSC samples were collected after 14 days of osteogenic differentiation. Briefly, RNA was extracted using TRIzol solution (Invitrogen). Then, RNA was quantified using a NanoDrop spectrophotometer and then subjected to reverse transcription to get cDNA. The cDNA was then mixed with dNTP, primers, and SyberGreen PCR Mix (Invitrogen) and amplified in a real-time quantitative polymerase chain reaction (PCR) instrument (Invitrogen) to detect the expression of targeted genes. The primer sequences are listed below (Table 1).

**TABLE 1:** Primers for PCR used in this study.

	Forward primer	Reverse primer
iNOS	TCC​CGA​GTC​AGA​GTC​ACC​AT	TCC​ATG​CAG​ACA​ACC​TTG​GG
TNF-α	GCC​CAT​GTT​GTA​GCA​AAC​CC	TGA​GGT​ACA​GGC​CCT​CTG​AT
CD163	CAG​CGG​CTT​GCA​GTT​TCC​TC	TGA​AAT​CAG​CTG​ACT​CAT​GGG​AA
CD206	GGG​AAA​GGT​TAC​CCT​GGT​GG	GTC​AAG​GAA​GGG​TCG​GAT​CG
Arg-1	TTC​TCA​AAG​GGA​CAG​CCA​CG	TAG​GGA​TGT​CAG​CAA​AGG​GC
GAPDH	CTA​GGC​GCT​CAC​TGT​TCT​CT	GCC​CAA​TAC​GAC​CAA​ATC​CGT
Runx2	CGC​CTC​ACA​AAC​AAC​CAC​AG	ACT​GCT​TGC​AGC​CTT​AAA​TGA​C
OSX	GTCCTTCTGAGGCGGCG	GCT​CCG​GTC​CTA​CAG​TCC​TA

### 2.4 Cell chemotaxis

#### 2.4.1 Interaction from ADSCs to macrophages

After coating the 64- and 0.2-kPa culture plates with collagen I, the ADSCs were inoculated into the plates and then grown until the density reached 60%. Transwell chambers with a pore size of 8 μm were selected and placed in a 24-well plate containing a macrophage differentiation medium. The THP-1 cells were seeded above the chamber to make the density reach 70% after adherence. The M0 macrophages were differentiated 24 h later and attached to the upper surface of the Transwell chambers. At this time, the chamber was taken out, washed briefly with PBS, and placed in 64- and 0.2-kPa culture plates inoculated with ADSCs. Standard culture plates inoculated with ADSCs were used as control. Besides, cell migration of macrophages in culture with ADSCs conditioned medium and normal culture medium was also performed. The plate was incubated in a constant-temperature incubator at 37°C for 12 h, and the migration of macrophages from the upper layer to the lower layer was detected. MDMs underwent the same treatment with THP-1-derived M0 macrophages for cell co-culture.

#### 2.4.2 Interaction from macrophages to ADSCs

The THP-1 cells were resuspended in the differentiation medium and seeded into 24-well plates, and the density of macrophages reached 60% after differentiation and adherence. After the differentiation was completed, the medium in the M0 group was replaced with a complete medium, whereas the media in the M1 and M2 groups were replaced with their respective polarized media. The plates were placed in an incubator for differentiation for 24 h, and then all the media were replaced with a complete medium. Transwell chambers (pore size 8 μm) were placed in an empty 24-well plate, and ADSCs were seeded on the upper layer of the chamber and grown until the density reached 70%. Then, the chambers inoculated with ADSCs were taken out, washed three times with PBS, and placed in a 24-well plate inoculated with macrophages of different polarization states. The entire culture system was placed in an incubator for 12 h, and the migration of ADSCs from the upper layer to the lower layer was detected. MDMs underwent the same treatment with THP-1-derived M0 macrophages.

#### 2.4.3 Crystal violet staining

After culturing for 12 h, the chambers were taken out from the culture plate and placed in a clean 24-well plate. The upper and lower surfaces of the chambers were gently washed three times with PBS. The chambers were placed in 4% paraformaldehyde solution and fixed at room temperature for 15 min. Then, the cells on the upper surface of the chambers were wiped clean, and the chambers were placed in the crystal violet staining solution for 30 min at room temperature. After staining, the chambers were taken out, rinsed with running water, and then air-dried at room temperature in a cool place, followed by macroscopic and microscopic observations of cell chemotaxis.

### 2.5 Effect of macrophages on the osteogenic differentiation of ADSCs

#### 2.5.1 Conditioned medium treatment

ADSCs were seeded into 12-well plates. The conditioned medium for three different macrophages was prepared (M0, M1, and M2). Subsequently, the conditioned medium was transferred to a complete osteogenic induction medium. The Normal Control Group (NC) used complete osteogenic induction medium without macrophage conditioned medium. ADSCs were washed three times with PBS solution, and then the medium was replaced with a mixed medium for osteogenic induction. The mixed medium was replaced every 3 days until the 14th day of osteogenic induction for subsequent characterization.

#### 2.5.2 Cell co-culture

ADSCs were seeded in 12-well plates. THP-1 cells were seeded into Transwell chambers (pore size 0.3 μm) and cultured with a differentiation medium for 24 h to differentiate them into M0 macrophages. Then, the medium was replaced with the corresponding M1 and M2 polarization-inducing solutions, and macrophages were cultured in a constant-temperature incubator for 24 h to make it polarized. The culture medium of ADSCs was replaced with an osteogenic differentiation medium, and the chambers containing different macrophages were placed in a 12-well plate to conduct osteogenic differentiation experiments of ADSCs under co-culture conditions with macrophages. Transwell chambers containing macrophages in 12-well plates were replaced every 3 days. The Transwell chambers were discarded after 14 days of osteogenic induction, and the osteogenically induced ADSCs in the culture plate were taken for subsequent characterization experiments. MDMs underwent the same treatment with THP-1-derived M0 macrophages.

#### 2.5.3 Alizarin red staining

The ADSCs after osteogenic induction were gently rinsed three times with Dulbecco’s phosphate-buffered saline (DPBS) solution without calcium and magnesium and then fixed with 4% paraformaldehyde solution at room temperature for 15 min. After fixation, ADSCs were rinsed with ultrapure water three times to remove residual paraformaldehyde. Then, 1% alizarin red staining (ARS) solution was prepared using Tris-HCl solution with a pH value of 8.3, and the ARS solution that could cover the bottom of the well was added to each well. The culture plate was placed on a shaking table at room temperature for 30 min. Then, the ARS solution was discarded and ADSCs were rinsed repeatedly with ultrapure water, followed by macroscopic and microscopic observation. For the quantitative analysis of ARS staining, cetylpyridine chloride solution was prepared using PBS solution with pH value of 7.2–7.4. After the microscopic observation of ARS staining, the cetylpyridine chloride solution was added into culture wells to dissolve calcium nodules stained with ARS. Then the absorbance of the solution above was measured at a wavelength of 562 nm.

#### 2.5.4 Alkaline phosphatase staining

The ADSCs after osteogenic induction were gently rinsed three times with the DPBS solution without calcium and magnesium. Then, 400 μL of the alkaline phosphatase (ALP) staining solution was added to each well, and the culture plate was placed in an incubator at 37°C and incubated in the dark for 15 min. The culture plate was taken out immediately after the incubation, the ALP staining solution was immediately discarded, and ultrapure water was immediately added to wash the cells three times to quickly terminate the reaction. Subsequent microscopic observation and photography were performed. ALP was quantified using Alkaline Phosphatase Assay Kit (Beyotime). Briefly, ADSCs underwent osteogenic differentiation for 7 days. Then cell lysate was extracted with the extraction buffer. 0.5 mM p-nitrophenol solution were added into cell lysate and incubated for 10 min at 37°C. The reaction was stopped by the stop buffer and the mixture was then measured at the wavelength of 405 nm.

### 2.6 Proteomic analysis

The conditioned medium of ADSCs and macrophages was filtered, concentrated, and then quantified using bicinchoninic acid assay (Invitrogen). The total protein was extracted using acetone and digested with trypsin. Then, the samples were subjected to LC-MS/MS and then analyzed with MaxQuant. The protein database was sourced from the UNIPROT database (Uniprot_human_2016_09). The protein sequence and its reverse decoy sequence were simultaneously used for MaxQuant library search. The quantitative type was non-standard quantification (LFQ) containing match between runs. Trypsin was set as a specific endonuclease, with a maximum of 3 missing sites; Oxidation [M] and Acetyl [protein N-term] were set as variable modifications. Carbamidomethyl [C] was fixed modifications, and the maximum number of variable modifications was 3. The FDR at both peptide and protein levels was 0.01, and the unique peptide without variable modification was used for quantification. Subsequently, the samples were standardized. After log conversion of LFQ quantitative results, the missing values were filled by random sampling from normal distribution using Perseus software, and the protein groups with non-missing values less than the number of sample duplicates were rounded off. Subsequently, statistical analysis was conducted on the standardized quantitative results to obtain corresponding differentially expressed proteins. We define proteins with a difference in expression fold change >1.5, unique peptide ≥2 and *p*-value <0.05 as proteins with significant differences. Then Gene Ontology (GO), Kyoto Encyclopedia of Genes and Genomes (KEGG) enrichment, and STRING analyses were conducted subsequently.

### 2.7 Enzyme linked immunosorbent assay (ELISA)

Cell conditioned medium was prepared and centrifuged at 2000 *g* for 10 min to discard cell debris. TNF-α, TGF-β, and LRP1 were quantified in the conditioned medium of macrophages in different polarization states. RBP4 was quantified in the conditioned medium of ADSCs on substrates with different stiffness. The quantification was according to the manufacturers’ instructions strictly. Briefly, conditioned medium was added into a 96-well plate which was pre-coated by the corresponding antibody. The samples were incubated in 37°C for 1 h and washed for 3 times. Then enzyme-linked antibodies were added and incubated for another 1 h. Reaction substrate was added into the plate subsequently and incubated at 37°C for 5 min protected from light. Then the stop solution was added immediately after incubation. The result was quantified by measuring absorbance within 20 min.

### 2.8 Statistical analysis

All experiments were performed at least independently and repeated three times. All data were expressed as mean ± standard deviation. Statistical difference was calculated using one-way ANOVA and a *p*-value < 0.05 represents statistically significant difference. * represents *p* < 0.05 *versus* control, ** represents *p* < 0.01 *versus* control, and *** represents *p* < 0.001 *versus* control.

## 3 Results

### 3.1 Effects of ADSCs on macrophage polarization

**FIGURE 1:**
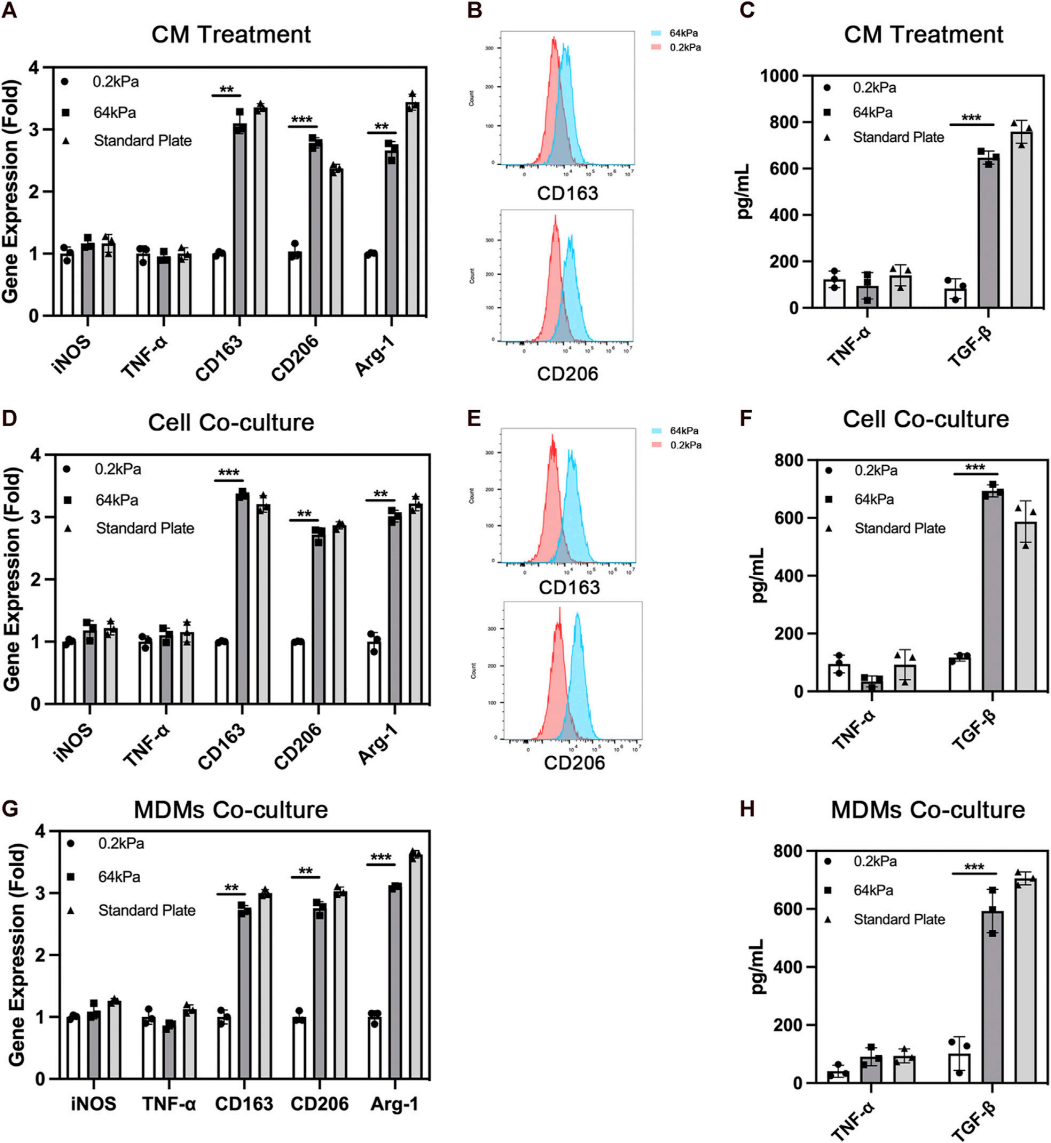
Polarization of macrophages under the influence of adipose-derived mesenchymal stem cells. **(A)** Real-time PCR **(B)** flow cytometry and **(C)** ELISA of macrophages with the treatment of conditioned medium from ADSCs on different substrates. **(D)** Real-time PCR **(E)** flow cytometry and **(F)** ELISA of macrophages co-cultured with ADSCs on different substrates. **(G)** Real-time PCR and **(H)** ELISA of MDMs co-cultured with ADSCs on different substrates. Data are presented as the means of three different replicates ±standard deviation. One-way ANOVA is performed to determine statistical differences. * represents *p* < 0.05 *versus* control, ** represents *p* < 0.01 *versus* control, and *** represents *p* < 0.001 *versus* control.

Presently, the commonly used absorbable biomaterials for bone repair in clinical practice, especially for non-weight-bearing bone repair, are mainly stiff biomaterials with Young’s modulus above more than 60 kPa. We selected stiff materials with Young’s modulus of 64 kPa for this study. A soft material with a modulus of 0.2 kPa was used as a negative reference. First, we explored the effect of ADSCs on the polarization of macrophages. We treated macrophages with a conditioned medium and cell co-culture. ADSCs were seeded on different substrates including 64 kPa, 0.2 kPa and standard culture plates to collect conditioned medium in the conditioned medium treatment. Then the different conditioned medium was used to stimulate THP-1-derived macrophages. The results showed that the expression of M2 polarization markers, CD163, CD206 and Arg-1, were significantly upregulated in macrophages in the 64-kPa group and standard plate group compared with the 0.2-kPa group (Figure 1A). There was no significant difference between 64-kPa group and standard plate group. Meanwhile, most macrophages were positive for CD163 and CD206 in the 64-kPa group (Figure 1B), indicating that the macrophages in the 64-kPa group underwent M2 polarization. ELISA found higher TGF-β secretion in macrophages of 64 kPa group (Figure 1C). Subsequently, we verified the aforementioned results by cell co-culture and obtained results with the same trend. ADSCs were seeded on different substrates in the lower chamber while THP-1-derived macrophages were seeded on the upper side of Transwell chamber. The expression levels of CD163, CD206 and Arg-1 were upregulated, and more cells were CD163 and CD206 positive in the 64-kPa group (Figures 1D, E). The higher secretion of TGF-β was also found by ELISA (Figure 1F). Besides, we used MDMs to further confirm the above results and found upregulation of CD163, CD206 and Arg-1. MDMs also had higher secretion of TGF-β (Figure 1H) These findings verified the immunoregulatory effect of ADSCs on stiff substrates, indicating the immune properties of stiff biomaterials.

### 3.2 Effects of ADSCs on the migration of macrophages

**FIGURE 2:**
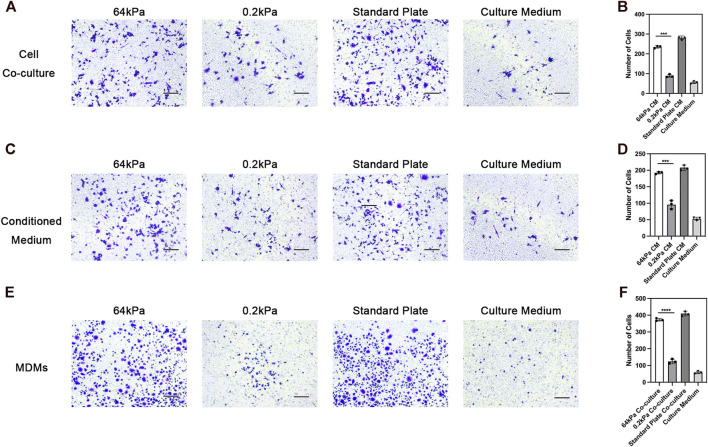
Chemotaxis of macrophages under the influence of adipose-derived mesenchymal stem cells. **(A)** Chemotaxis of THP1-derived macrophages co-cultured with ADSCs in the lower chamber and **(B)** quantification. **(C)** Chemotaxis of THP1-derived macrophages in culture with ADSCs conditioned medium and **(D)** quantification. **(E)** Chemotaxis of MDMs co-cultured with ADSCs in the lower chamber and **(F)** quantification. Data are presented as the means of three different replicates ±standard deviation. One-way ANOVA is performed to determine statistical differences. * represents *p* < 0.05 *versus* control, ** represents *p* < 0.01 *versus* control, and *** represents *p* < 0.001 *versus* control. Scale bar = 50 μm.

Chemotaxis is one of the most important biological behaviors of macrophages. We used the Transwell assay to evaluate the effect of ADSCs on the chemotactic ability of macrophages on the substrates with different stiffness. ADSCs were seeded on different substrates including 64 kPa, 0.2 kPa and standard culture plates in the lower chamber while THP-1-derived macrophages were seeded on the upper side of Transwell chamber (Figures 2A, B). Besides, cell migration of macrophages in culture with ADSCs conditioned medium and normal culture medium was also performed (Figures 2C, D). We found that significantly more cells were stained with crystal violet in the lower layer of the Transwell chamber in the 64-kPa group, indicating that the ADSCs in the 64-kPa group had a stronger effect on the migration of macrophages, which represented a stronger potential to recruit circulating macrophages. Furthermore, we used MDMs to perform the macrophage migration experiments above and got similar results (Figures 2E, F). This part proved that M2 macrophages had stronger potential to recruit circulating macrophages.

### 3.3 Analysis of secreted proteins from ADSCs

**FIGURE 3:**
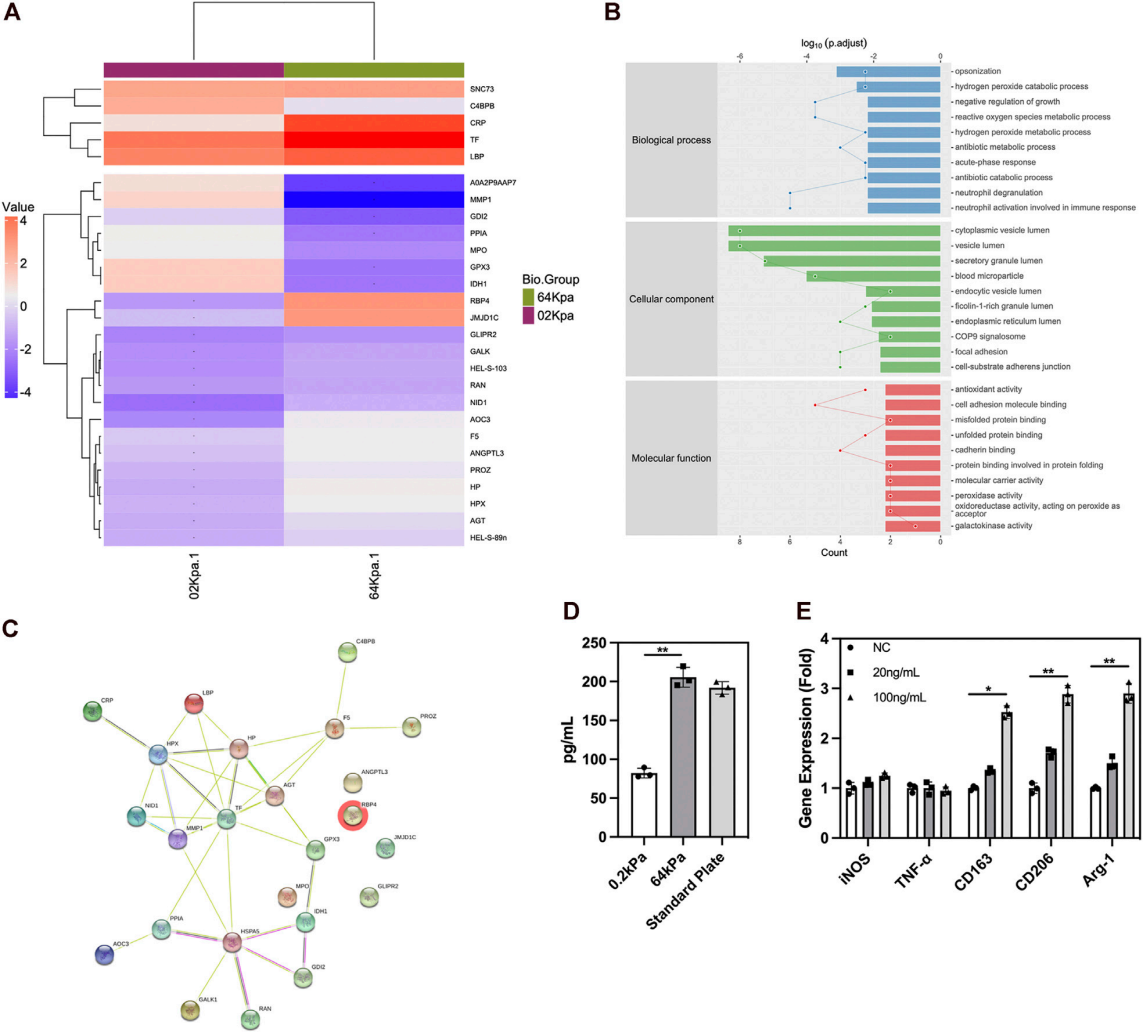
Protein secretion of adipose-derived mesenchymal stem cells on different substrates. **(A)** Protein secretion heatmap of ADSCs on different substrates. **(B)** GO analysis of differentially expressed proteins. **(C)** STRING protein interaction prediction of differentially expressed proteins. **(D)** ELISA of RBP4 in ADSCs conditioned medium. **(E)** PCR analysis of polarization genes in macrophages under the influence of RBP4. Proteins with a difference in expression fold change >1.5, unique peptide ≥2 and *p*-value <0.05 were defined as proteins with significant differences.

We conducted mass spectrometry analysis on the secreted proteins of ADSCs on biomaterials with different stiffness to clarify the underlying mechanism. ADSCs are adult stem cells with weak protein secretion capacity compared with immune cells. We detected 395 proteins in total, and 27 proteins exhibited significant differences in expression. Among these differentially expressed proteins, the types and abundance of secreted proteins of ADSCs on the surface of 64-kPa materials significantly increased, and they could secrete immune-activating proteins such as TF, CRP, LBP, and RBP4 (Figure 3A). These proteins had the potential to recruit and activate macrophages. The GO enrichment analysis found that the differential protein secretion between the two groups was mainly enriched in immune activation function, protein synthesis, and transport function (Figure 3B), which are the main characteristics of immune regulatory proteins. Meanwhile, the prediction of STRING protein interaction revealed that the protein interaction network centered on immunomodulatory proteins such as TF, LBP, and RBP4 (Figure 3C), which indicated the pivotal role of these proteins in the immune properties of ADSCs, indicating that TF, LBP, and RBP4 might be the effector proteins secreted by ADSCs to induce macrophage polarization and chemotaxis. The conclusions about immune cell status and function were consistent. Furthermore, we validated the proteomics result with ELISA and found higher RBP4 secretion in ADSCs on 64 kPa substrates than those on 0.2 kPa substrates (Figure 3D). RBP4 could induce M2 polarization of macrophages (Figure 3E). The aforementioned results confirmed that ADSCs on the surface of the 64-kPa biomaterials could secrete more immunomodulatory proteins, thus affecting the biological function of macrophages and making them undergo M2 polarization. Proteomic analysis preliminarily unraveled the underlying mechanism of this process and found potential regulatory proteins.

### 3.4 Effects of macrophages on the osteogenic differentiation of ADSCs

**FIGURE 4:**
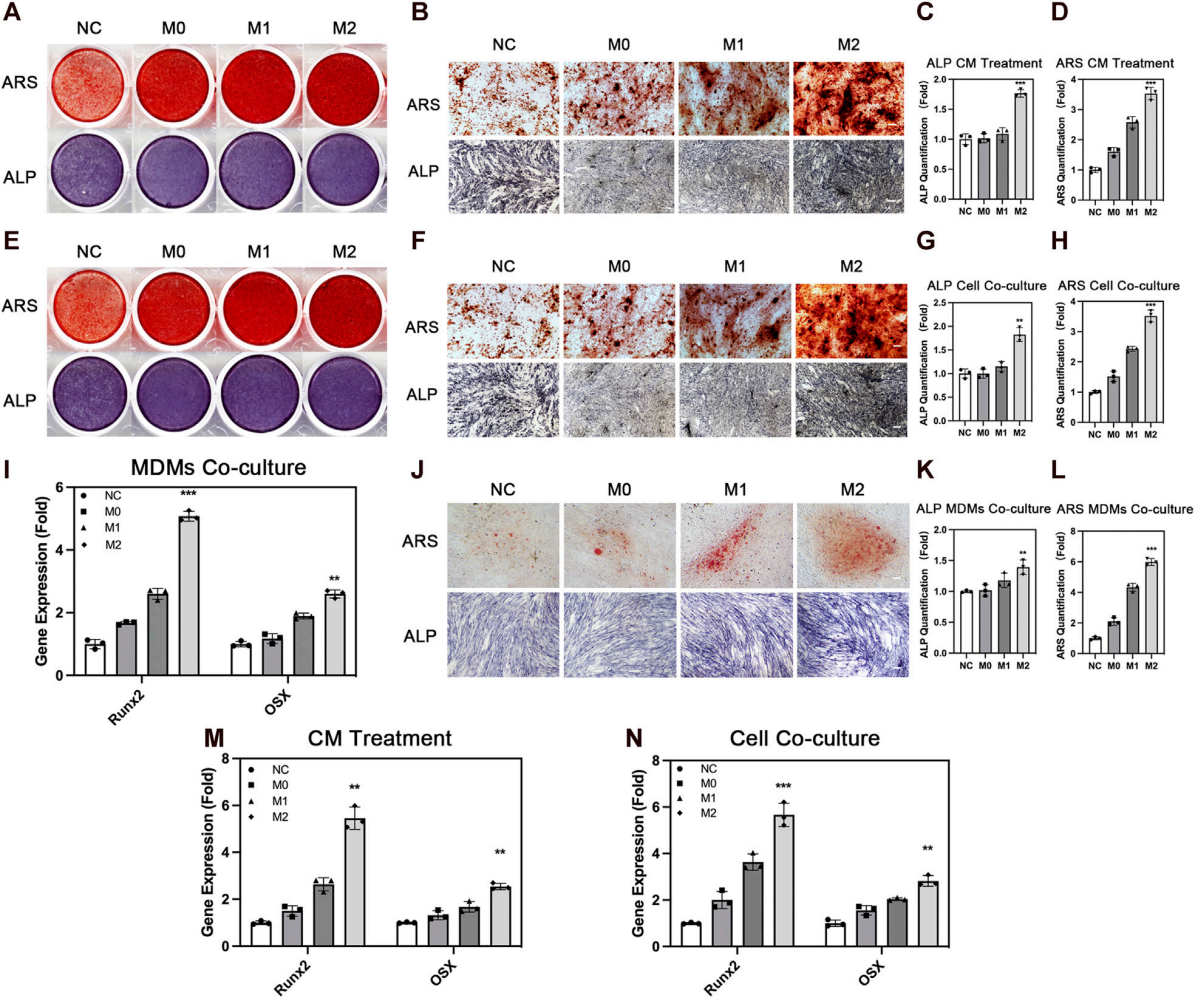
Osteogenic differentiation of adipose-derived mesenchymal stem cells under the influence of macrophages. **(A)** Overall picture and **(B)** microscopic image of ARS and ALP staining of ADSCs with the treatment of conditioned medium from THP1-derived macrophages in different polarization states. **(C)** Quantification of ALP and **(D)** ARS under conditioned medium treatment. **(E)** Overall picture and **(F)** microscopic image of ADSCs co-cultured with THP1-derived macrophages in different polarization states. **(G)** Quantification of ALP and **(H)** ARS in cell co-culture. **(I)** Runx2 and OSX expression in ADSCs co-cultured with MDMs in different polarization states. **(J)** Microscopic image of ARS and ALP staining of ADSCs co-cultured with MDMs in different polarization states. **(K)** Quantification of ALP and **(L)** ARS in MDMs co-culture. **(M)** Runx2 and OSX expression in ADSCs with the treatment of conditioned medium from THP1-derived macrophages in different polarization states and **(N)** co-cultured with THP1-derived macrophages in different polarization states. Data are presented as the means of three different replicates ±standard deviation. One-way ANOVA is performed to determine statistical differences. * represents *p* < 0.05 *versus* control, ** represents *p* < 0.01 *versus* control, and *** represents *p* < 0.001 *versus* control. Scale bar = 50 μm.

ADSCs affect the behavior of macrophages. However, macrophages also conversely affect the biological behavior of ADSCs, thereby affecting bone repair. Therefore, we explored the effect of the different polarization states of macrophages on the osteogenic differentiation of ADSCs. We treated ADSCs with a conditioned medium and cell co-culture and evaluated the osteogenic differentiation of ADSCs. We evaluated the osteogenic differentiation of ADSCs in the conditioned medium treatment group. ALP staining showed that the deep staining degree of ALP in the M2 group was the highest among the four groups after 7 days of conditioned medium treatment and osteogenic induction (Figures 4A–C). The quantitative results of ALP expression were also in line with the aforementioned results. The results of ARS showed that the number and area of calcium nodules formed in ADSCs were in the sequence of M2 group > M1 group > M0 group > NC group (Figures 4A, B, D) after 14 days of conditioned medium treatment and osteogenic induction. The quantitative results were also in line with this trend.

We used the cell co-culture method to verify the aforementioned results and to create a more realistic processing condition to truly reflect the real crosstalk of cells. The results of ALP staining and ARS were consistent with this trend under the condition of cell co-culture (Figures 4E–H). The three groups under the influence of macrophages showed better osteogenic differentiation effect compared with the NC group without macrophage conditioned medium or macrophage co-culture, indicating that the participation of macrophages promoted the osteogenesis of ADSCs. Among the macrophages in three different polarization states, M2 macrophages had a stronger promoting effect on osteogenic differentiation, which was consistent with the repair-promoting properties of M2 macrophages. Osteogenic genes including Runx2 and OSX were also evaluated and their expression was significantly higher in M2 group than the other groups (Figures 4M, N). Besides, we used MDMs to better confirm the results above. M2 macrophages showed stronger osteogenic promoting capacity in ALP and ARS staining evaluation, which was in line with the results from THP-1-derived macrophages (Figures 4I, L). In summary, these findings showed the importance of macrophage participation in bone repair, especially M2 macrophages with strong osteogenesis-promoting capacity.

### 3.5 Effects of macrophages on the migration of ADSCs

**FIGURE 5:**
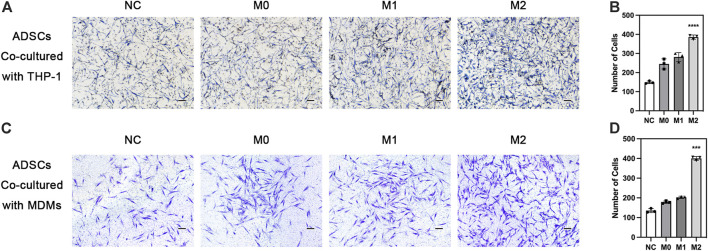
Chemotaxis of adipose-derived mesenchymal stem cells under the influence of macrophages. **(A)** Chemotaxis of ADSCs co-cultured with THP1-derived macrophages in different polarization states and **(B)** quantification. **(C)** Chemotaxis of ADSCs co-cultured with MDMs in different polarization states and **(D)** quantification. vData are presented as the means of three different replicates ±standard deviation. One-way ANOVA is performed to determine statistical differences. * represents *p* < 0.05 *versus* control, ** represents *p* < 0.01 *versus* control, and *** represents *p* < 0.001 *versus* control. Scale bar = 50 μm.

The chemotactic function of the local stem cells is also extremely important in bone defect repair, besides the chemotactic function of macrophages. Exogenous stem cells on biomaterials must migrate to the ossification center for differentiation. Meanwhile, endogenous stem cells from the circulation need to migrate to the injury site to enrich the local stem cell pool. We explored the effect of macrophages in different polarization states on the chemotactic ability of ADSCs. THP-1-derived macrophages in different polarization states were seeded in the lower chamber while ADSCs were seeded on the upper side of Transwell chamber. The results showed that ADSCs in the other three groups inoculated with macrophages were more favorable to migrate to macrophages than the cells in the NC group without macrophages, indicating that macrophages could recruit mesenchymal stem cells. The comparison of the M0, M1, and M2 groups revealed that the macrophages in the M2 group could significantly enhance the chemotactic ability of ADSCs, which was significantly higher than those in the M1 and M0 groups, evaluated using crystal violet staining (Figures 5A, B). Besides, we used MDMs to perform the ADSCs migration experiments above and got similar results (Figures 5C, D). The results indicated that M2 macrophages could enhance the chemotactic ability of stem cells nearby and had stronger potential to recruit circulatory stem cells, which was of great value to bone regeneration.

### 3.6 Analysis of secreted proteins from macrophages

**FIGURE 6:**
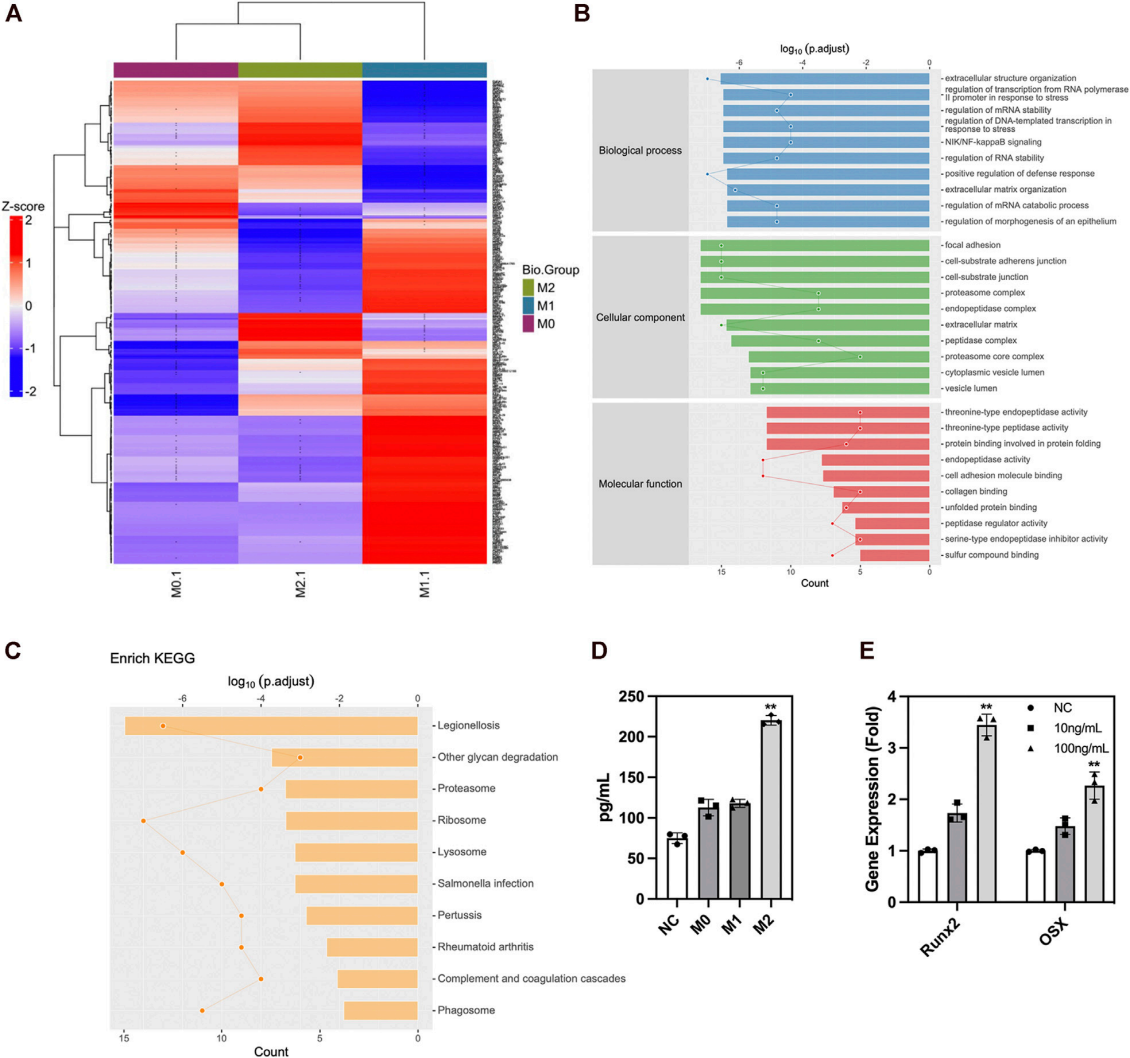
Protein secretion of macrophages in different polarization states. **(A)** Protein secretion heatmap of macrophages in different polarization states. **(B)** GO analysis of differentially expressed proteins. **(C)** KEGG pathway enrichment of differentially expressed proteins. **(D)** ELISA of LRP1 in macrophage conditioned medium. **(E)** PCR analysis of osteogenic genes in ADSCs under the influence of LRP1. Proteins with a difference in expression fold change >1.5, unique peptide ≥2 and *p*-value <0.05 were defined as proteins with significant differences.

**FIGURE 7:**
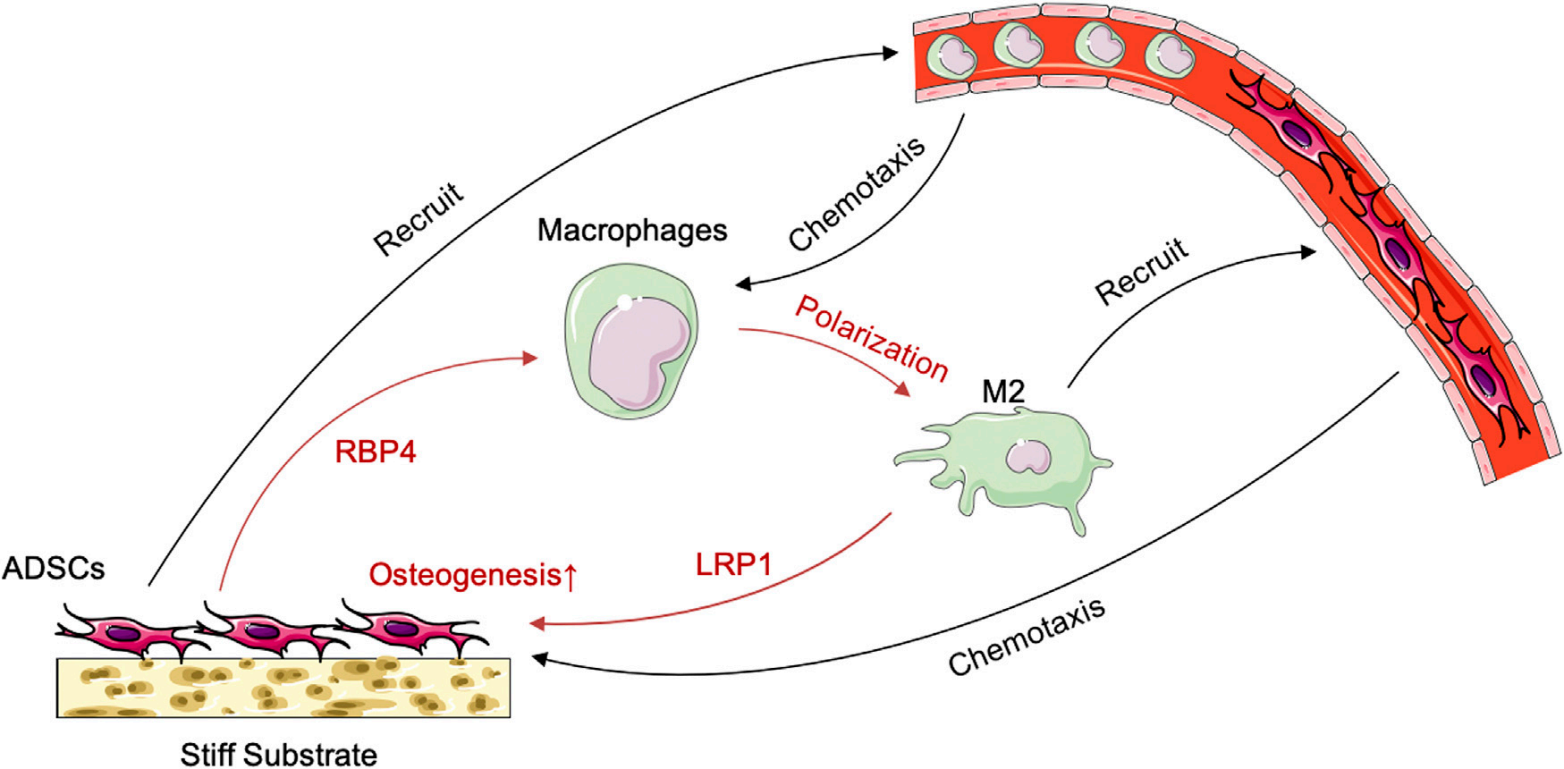
Schematic illustration of the crosstalk between ADSCs and macrophages under the influence of stiff biomaterials.

We analyzed the secreted protein profiles of macrophages in different polarization states to clarify the possible mechanism by which macrophages affected the biological function of ADSCs. Macrophages are active immune cells with strong secreting capacity. We detected 812 proteins and found 230 differentially expressed proteins between M2 and M1 macrophages, 217 differential proteins between M1 and M0 macrophages, and 90 proteins between M2 and M0 macrophages. Among the secreted proteins from the three types of macrophages, M1 macrophages exhibited the highest number and variety of secreted proteins showing a significantly greater difference than the secreted protein profiles of M0 and M2 macrophages. The M1 macrophages mainly secreted a large number of immune-activating proteins, which mediated intense immune responses against pathogens or damaged tissues and corresponded to their role in the M1–M2 sequential polarization in bone repair. Among the secreted proteins of macrophages, those of M2 macrophages were mainly LRP1 and other proteins with the ability to promote tissue repair. These proteins could exert their effect by creating an immune microenvironment suitable for tissue repair to promote tissue regeneration (Figure 6A). The GO enrichment analysis of cell function showed that the M1 macrophage–secreted proteins were mainly proinflammatory proteins to activate immune cells such as neutrophils and enhance the production and secretion of proinflammatory cytokines such as IFN-γ, IL-6, and TNF-α. However, the functions of M2 macrophage–secreted proteins mainly included cell adhesion, protein and polysaccharide synthesis, and the secretion and remodeling of extracellular matrix, which is of great importance to tissue repair. Among those functions, extracellular matrix remodeling had the strongest correlation with bone repair (Figure 6B). The KEGG pathway enrichment also found the activation of the ribosome, lysosome, and proteasome-related pathways, which were compatible with the biological function of extracellular matrix remodeling (Figure 6C). Besides, we performed ELISA to validate the proteomics result and found higher LRP1 secretion in M2 macrophages (Figure 6D). LRP1 showed osteogenic promoting capacity as PCR showed (Figure 6E). In summary, these findings revealed that M2 macrophages could effectively promote the secretion and remodeling of extracellular matrix, which initially explained the reason why M2 macrophages promoted the osteogenic differentiation of ADSCs. Figure 7.

## 4 Discussion

Biomaterials are critical elements in bone tissue engineering. Traditionally, biomaterials with high stiffness are preferred for mimicking the biological strength of bone to imitate physiological repair. Few studies considered other potential advantages of this choice. With the development of materials science, the inherent properties of biomaterials could be adjusted precisely, thus providing more parameters for biomaterial research. In previous studies, researchers mainly focused on the structural and surface engineering of biomaterials, thereby evaluating the influences of elasticity, stiffness, appearance, and structure on their biological effects. The rapid development of immunology brought new insights into biomaterial design and manufacturing, giving rise to a series of scientific research on the immunological properties of biomaterials and the pivotal roles of inherent properties, which is a crucial issue in this field deciding the tissue adaptability and repair effect of biomaterials. Currently, the relationship between the immunological properties and the inherent properties of biomaterials has not been fully explained. This may still be the hottest issues shedding new light on this field.

The immunological properties of biomaterials have an important impact on the effect of bone repair. In this study, we investigated the effect of the stiffness of biomaterials on their immunological properties, a scientific issue that has not been studied clearly yet. We found unique crosstalk between ADSCs and macrophages on stiff substrates. Compared with ADSCs on the 0.2-kPa substrate, ADSCs on the 64-kPa substrate could secrete more immunoregulatory proteins, such as LBP and RBP4 (Figure 3), to promote the M2 polarization of macrophages (Figure 1), enhancing the chemotaxis of local macrophages (Figure 2), and recruit circulatory macrophages. Meanwhile, M2 macrophages could secrete extracellular matrix–remodeling proteins such as LRP1 (Figure 6) to promote the osteogenic differentiation of ADSCs (Figure 4), enhancing the chemotaxis of local endogenous stem cells to enrich the local stem cell pool (Figure 5). Besides clarifying this crosstalk, the present study also analyzed the expression profiles of the secreted proteins of ADSCs and macrophages through proteomic analysis, comprehensively demonstrating the immunoregulatory function of ADSCs and macrophages, which was the basis for the immune regulation of bone repair materials. This study provided a new understanding of the immunological properties of biomaterials.

In bone tissue engineering, most exogenous stem cells and some immune cells adhere to the surface of biomaterials. The inherent properties of the biomaterials, such as stiffness and surface morphology (Kyle and David, 2017; Ming et al., 2017; Jessica et al., 2018), affect the behavior of cells. Immune cells have shown different phenotypes of pro-inflammatory or anti-inflammatory response to different substrate stiffness in different studies. Previous studies revealed the direct effect of biomaterial stiffness on macrophages attached to the biomaterial (Joan et al., 2021; Yizhou et al., 2021; Hemant et al., 2022), which revealed the variable effect of substrate stiffness. However, in the repair microenvironment, most immune cells are inherent to play their roles in the tissue (Erika et al., 2021) and are hardly directly affected by biomaterials. Therefore, this study focused on the protein secretion of ADSCs on the surface of biomaterials with different stiffness (Figure 3) and explored its effect on the polarization state of macrophages (Figure 2), revealing the regulatory effect of biomaterial immune properties on the repair microenvironment from another perspective. This study found that the immune-activating proteins, such as LBP and RBP4, could promote the M2 polarization of macrophages (Figure 3). Unlike IFN-γ, these proteins were moderate immune-activating proteins that did not produce intense immune responses and mainly promoted the repair process (Gentek et al., 2014). Moreover, previous studies showed that RBP4 could induce moderate inflammation in adipose tissue (Pedro et al., 2014). This study also explained the immunoregulatory role of these proteins in bone repair. In this section, we used ADSCs inoculated on normal culture plates as control group and found that the result was similar to that of the 64 kPa group with no significant difference between those two groups. We suppose that there is a certain range of substrate stiffness to which ADSCs could response. The protein secretion of ADSCs will undergo significant changes accordingly when substrate stiffness changes within a certain range. When the substrate stiffness exceeds the certain range, the protein secretion of ADSCs tends to stabilize and no significant changes occur. The Young’s modulus of the normal culture plate far exceeds 64kPa, which may have already exceeded the response range. ADSCs on normal culture plates may have protein secretion similar to those on other stiff substrates including 64 kPa. We are currently unable to determine the above-mentioned range of substrate stiffness in this study. More detailed substrate stiffness grouping is needed for further research.

Different polarization states of macrophages were key points of immunoregulation in bone repair, among which the M2 polarization is the most important promoter of the repair process (Pajarinen et al., 2019). Many previous studies revealed various mechanisms by which M2 macrophages promoted osteogenic differentiation and bone repair directly by activating osteogenic pathways (Min et al., 2022; Yaping et al., 2022), including the macrophage-derived osteogenic protein LRP1 (Linda et al., 2018), which we also found in proteomic analysis. This study found that M2 macrophages mainly secreted extracellular matrix–remodeling proteins to promote the osteogenic differentiation of ADSCs (Figures 4, 6), which was a different view from previous studies and explained a different role of proteins such as LRP1. The promotion of extracellular matrix remodeling is one of the typical characteristics of M2 macrophages (Shapouri-Moghaddam et al., 2018; Yunna et al., 2020), which has been elucidated in many studies on fibrotic diseases such as renal fibrosis and pulmonary fibrosis (Qi et al., 2015; Peiyong et al., 2021). However, not only extracellular matrix remodeling has the adverse effect of promoting fibrotic disease progression, but it is also a key process of osteogenic differentiation (Andrea et al., 2007). As soon as the osteogenic differentiation pathway of stem cells is activated, the stem cells begin to arrange in regular bundles. Also, pathways related to ribosomes, lysosomes, and proteasomes are activated (Figure 6), producing a large amount of extracellular matrix containing mucopolysaccharide and chondroitin sulfate (Caroline et al., 2014). The extracellular matrix continuously accumulates and remodels into regular bundles, forming the rudimentary bone microstructure. Then, calcium ions accumulate and cell calcification occurs (Fanxin, 2011). Finally, a mature bone with osteocytes and calcified matrix is formed. The secretion and remodeling of the extracellular matrix is the pivotal process of osteogenic differentiation, which provides a new explanation for the mechanism of M2 macrophages to promote bone repair. It also provides a new understanding of how stiff biomaterials promote bone regeneration.

We also found different trends in ALP and ARS staining (Figure 4). There was no significant difference in ALP between the M1 group and control or M0, but the ARS results showed M1 was better than control and M0. M1 and M0 group had higher cell fusion, more extracellular matrix secretion, and denser staining in ALP staining compared with NC group, which might be related to higher proliferation and matrix-secreting capacity and may affect the late stages of osteogenic differentiation and calcium deposition. M1 macrophages could secrete a series of pro-inflammatory cytokines, which can have different effects on osteogenesis at different concentrations or in different stages of osteogenesis. We suppose that the reason for this result may be that some inflammatory factors produced by M1 macrophages did not strongly promote osteogenic gene expression in the early stage of differentiation (from mesenchymal stem cells to osteoblast precursor cells), but promoted ADSCs proliferation or extracellular matrix secretion. The higher cell density and extracellular matrix amount had a positive impact on the differentiation process or calcium deposition process in the late stage of differentiation (from osteoblast precursor cells to osteoblasts and osteocytes), thus making calcium nodules significantly higher than those of control and M0 groups. Further research is needed on the key proteins and their mechanisms.

This study clarified the potential mechanism of stiff biomaterials to promote bone repair by regulating ADSC–macrophage crosstalk. Still, several key issues remain unaddressed. First, we used a standardized cell line THP-1 to conduct most experiments including proteomics to get data with high universality. MDMs were only used to demonstrate the rationality of our obtained data considering that MDMs from different donors had significant heterogeneity in function and phenotype. The secretory protein from MDMs should be systematically analyzed using large sample size to get more solid results, which is necessary in further study. Second, this study was limited to *in vitro* experiments. Hence, further *in vivo* experimental verification of *in situ* bone repair is needed to fully verify the correctness of the ADSC–macrophage crosstalk. Third, this study only clarified the underlying molecular mechanism of this crosstalk. Molecular biology experiments are still necessary to verify the key effector molecules and signaling pathways. These in-depth investigations might supply detailed evidence to guide further application in bone tissue engineering. Moreover, the existing and follow-up research might provide more scientific guidance on biomaterial development and clinical practice. The structure, surface topography, and stiffness of biomaterials should be considered comprehensively to maximally construct a better immune microenvironment, which would accelerate the bone repair process and achieve a better therapeutic effect.

## 5 Conclusion

In this study, we found that ADSCs on stiff biomaterials could secrete immunomodulatory proteins such as RBP4 to promote M2 polarization and enhance the chemotaxis of macrophages. Meanwhile, M2 macrophages could secrete extracellular matrix–remodeling proteins such as LRP1 to promote the osteogenic differentiation of ADSCs and enhance the chemotactic capacity of stem cells. This study provided a new understanding of the immunological properties of biomaterials for bone repair.

## Data Availability

The raw data supporting the conclusions of this article will be made available by the authors, without undue reservation.
